# Supplementary material for: System Theoretical Study on the Effect of Variable Nonmetallic Doping on Improving Catalytic Activity of 2D-Ti3C2O2 for Hydrogen Evolution Reaction
Source: Nanomaterials (Basel). 2021 Sep 25;11(10):2497. doi: 10.3390/nano11102497 (PMC8539186; doi:10.3390/nano11102497)
Supplement: Supplementary file 1 [file nanomaterials-11-02497-s001.zip › nanomaterials-1370662-supplementary.pdf]

Supporting Information

System Theoretical Study on the Effect of Variable Nonmetallic Doping on Improving Catalytic Activity of 2D-$Ti_3C_2O_2$ for Hydrogen Evolution Reaction

Ye Su †, Minhui Song †, Xiaoxu Wang *, Jihang Jiang, Xiaolong Si, Tianhang Zhao and Ping Qian *

Beijing Advanced Innovation Center for Materials Genome Engineering, School of Mathematics and Physics, , University of Science and Technology Beijing, Beijing 100083, China; suyechina@163.com (Y.S.); mhuisong@163.com (M.S.); xuying13520506589@163.com (J.J.); sixiaolong2018@outlook.com (X.S.); ztx13520805687@163.com (T.Z.)

* Correspondence: wxx18811349251@163.com (X.W.); pingqian@ustb.edu.cn (P.Q.)

† Ye Su and Minhui Song are co-first authors.

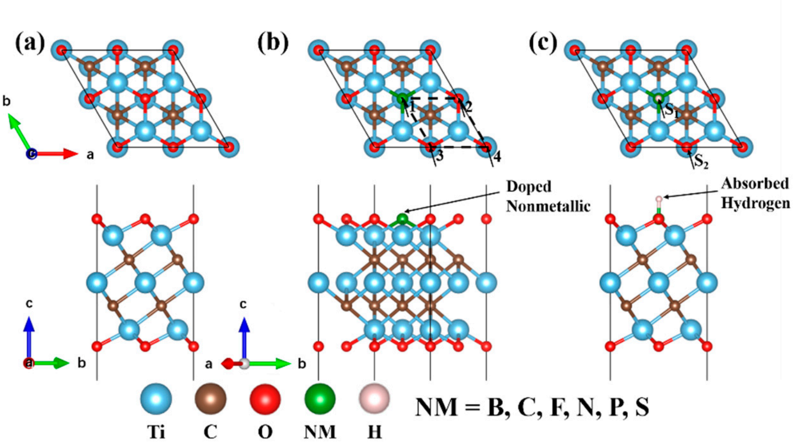

Figure S1. 0.25-1.00 concentrations nonmetallic element doping configurations.

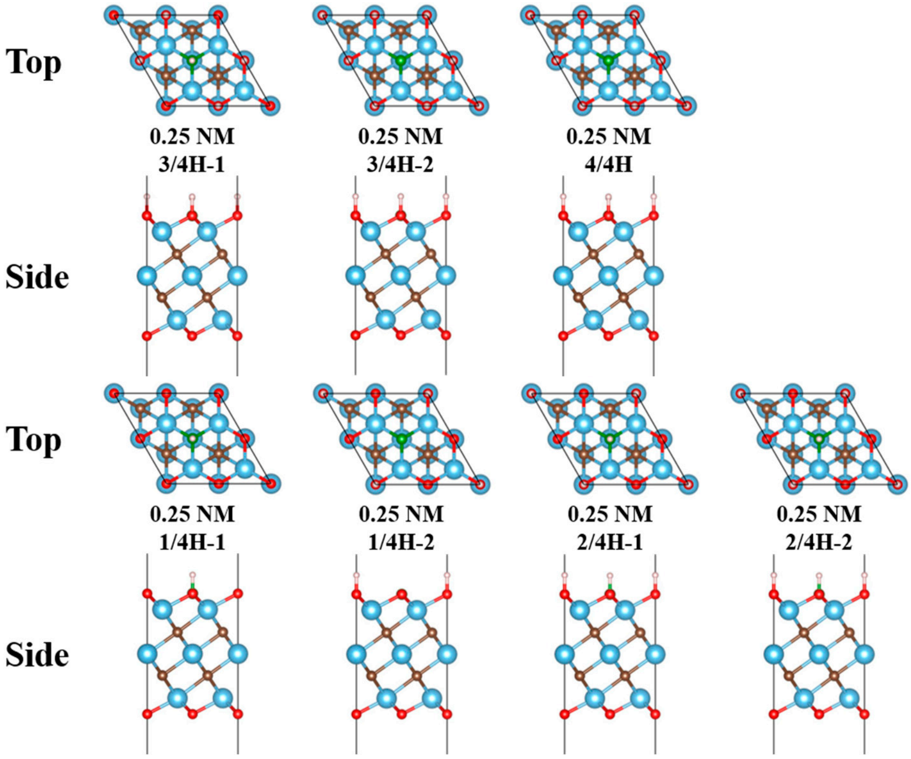

Top
0.25 NM 3/4H-1
0.25 NM 3/4H-2
0.25 NM 4/4H
Side
Top
0.25 NM 1/4H-1
0.25 NM 1/4H-2
0.25 NM 2/4H-1
0.25 NM 2/4H-2
Side

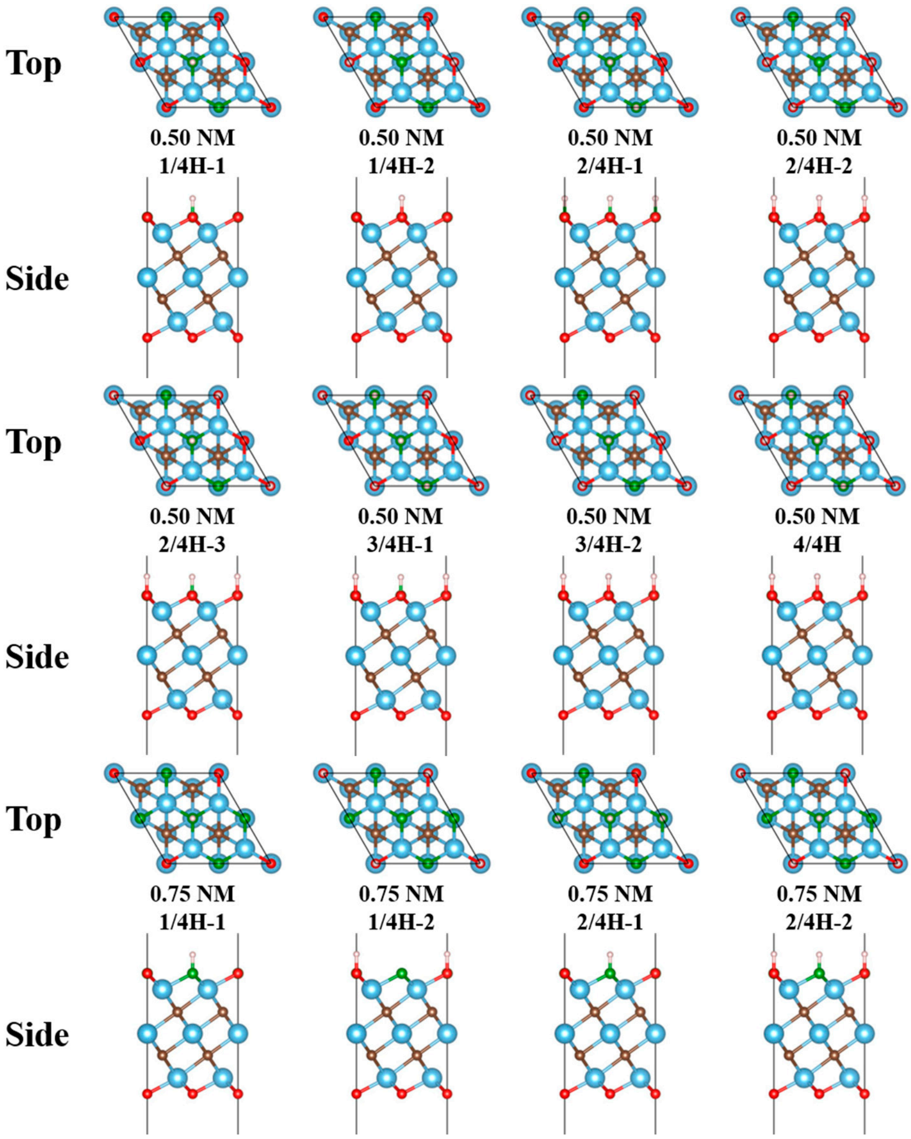
Top
Side
Top
Side
Top
Side
0.50 NM 1/4H-1
0.50 NM 1/4H-2
0.50 NM 2/4H-1
0.50 NM 2/4H-2
0.50 NM 2/4H-3
0.50 NM 3/4H-1
0.50 NM 3/4H-2
0.50 NM 4/4H
0.75 NM 1/4H-1
0.75 NM 1/4H-2
0.75 NM 2/4H-1
0.75 NM 2/4H-2

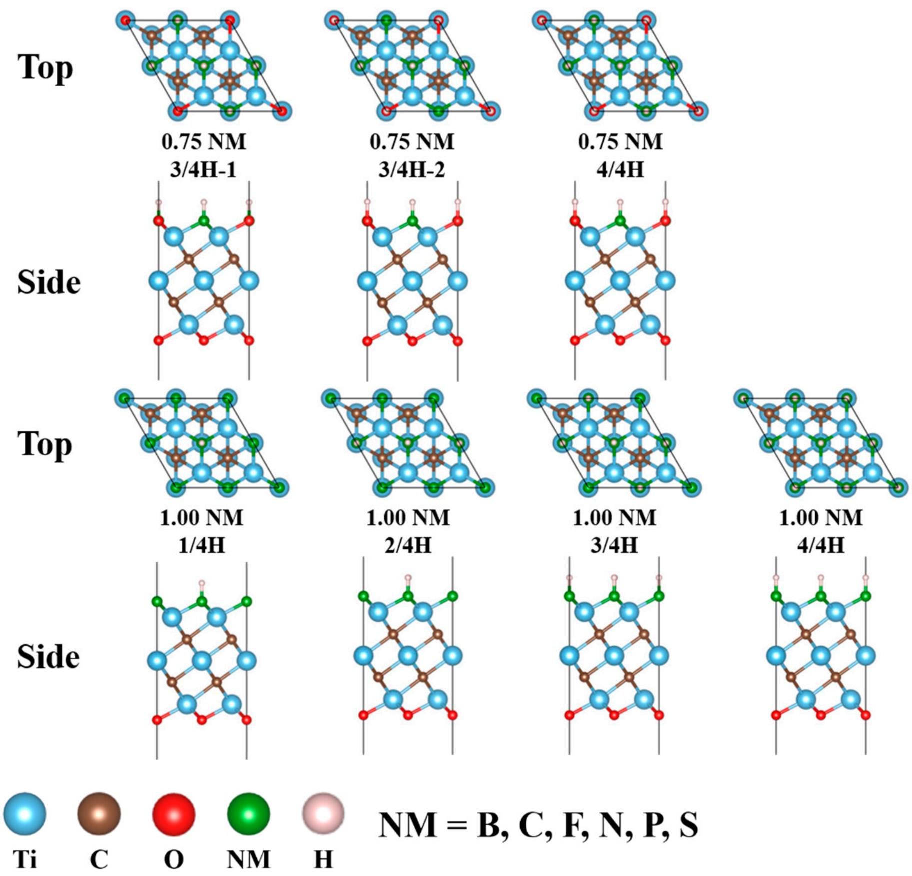

Figure S2. Different concentrations nonmetallic element doping at different H absorption concentrations configurations.

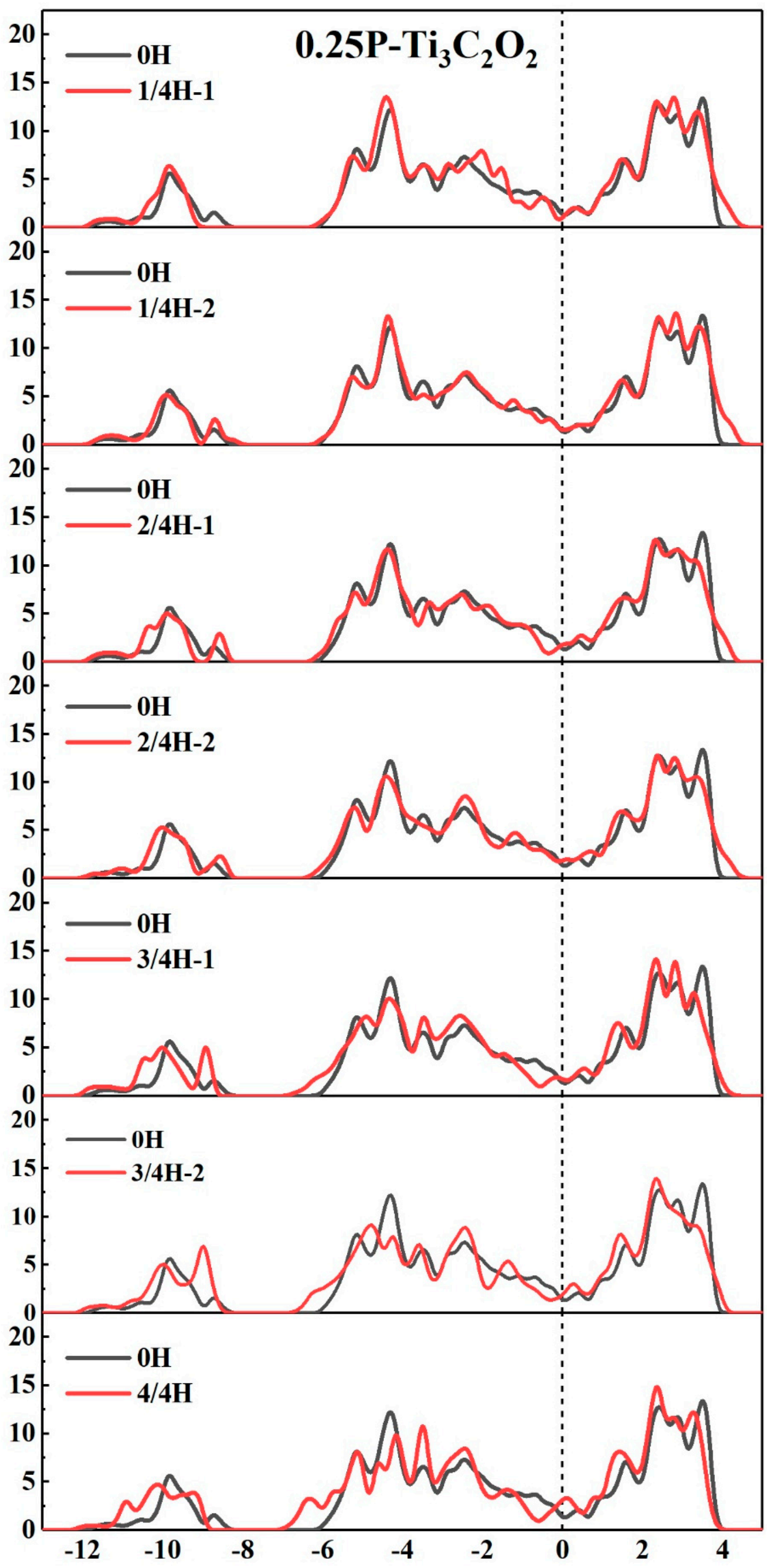

0.25P-Ti3C2O2
0H
1/4H-1
0H
1/4H-2
0H
2/4H-1
0H
2/4H-2
0H
3/4H-1
0H
3/4H-2
0H
4/4H
20
15
10
5
0
-12
-10
-8
-6
-4
-2
0
2
4

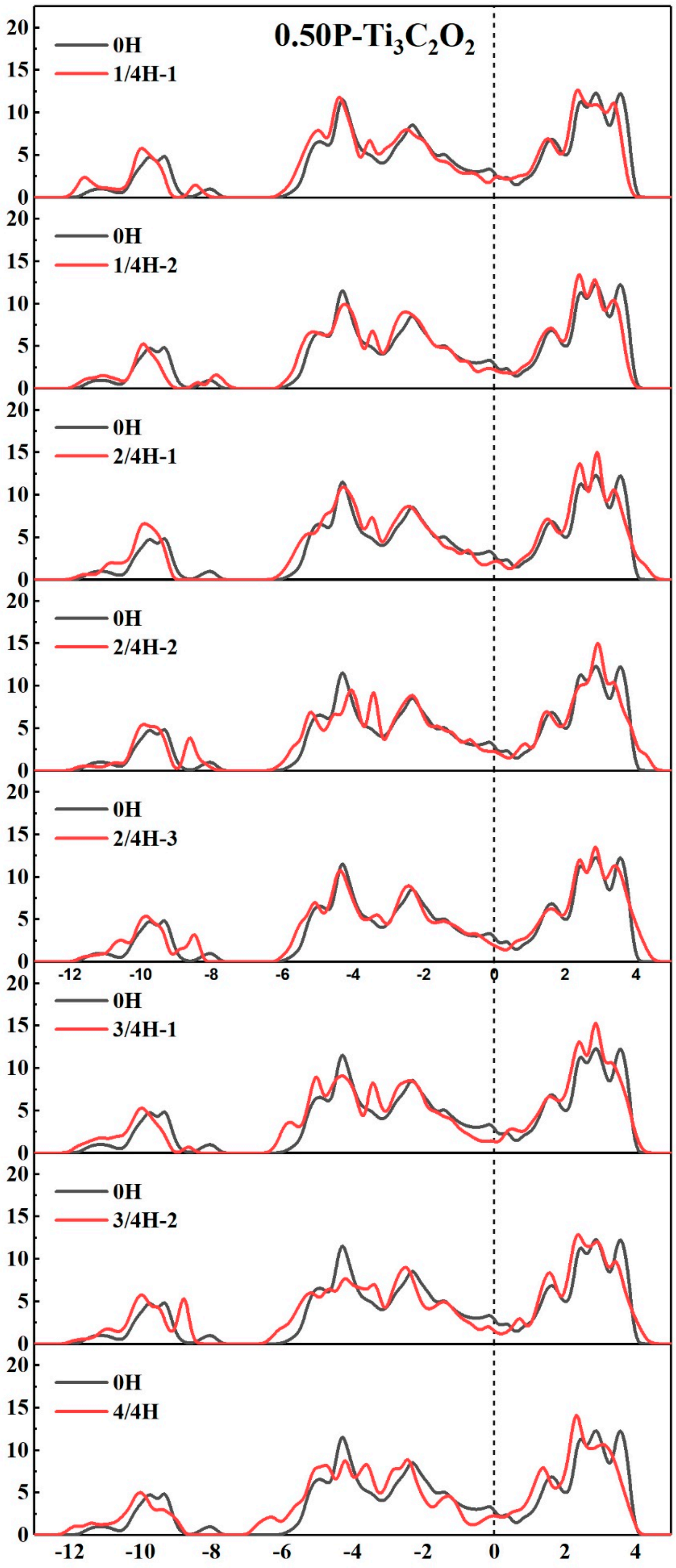

0.50P-$Ti_3C_2O_2$
0H
1/4H-1
0H
1/4H-2
0H
2/4H-1
0H
2/4H-2
0H
2/4H-3
0H
3/4H-1
0H
3/4H-2
0H
4/4H

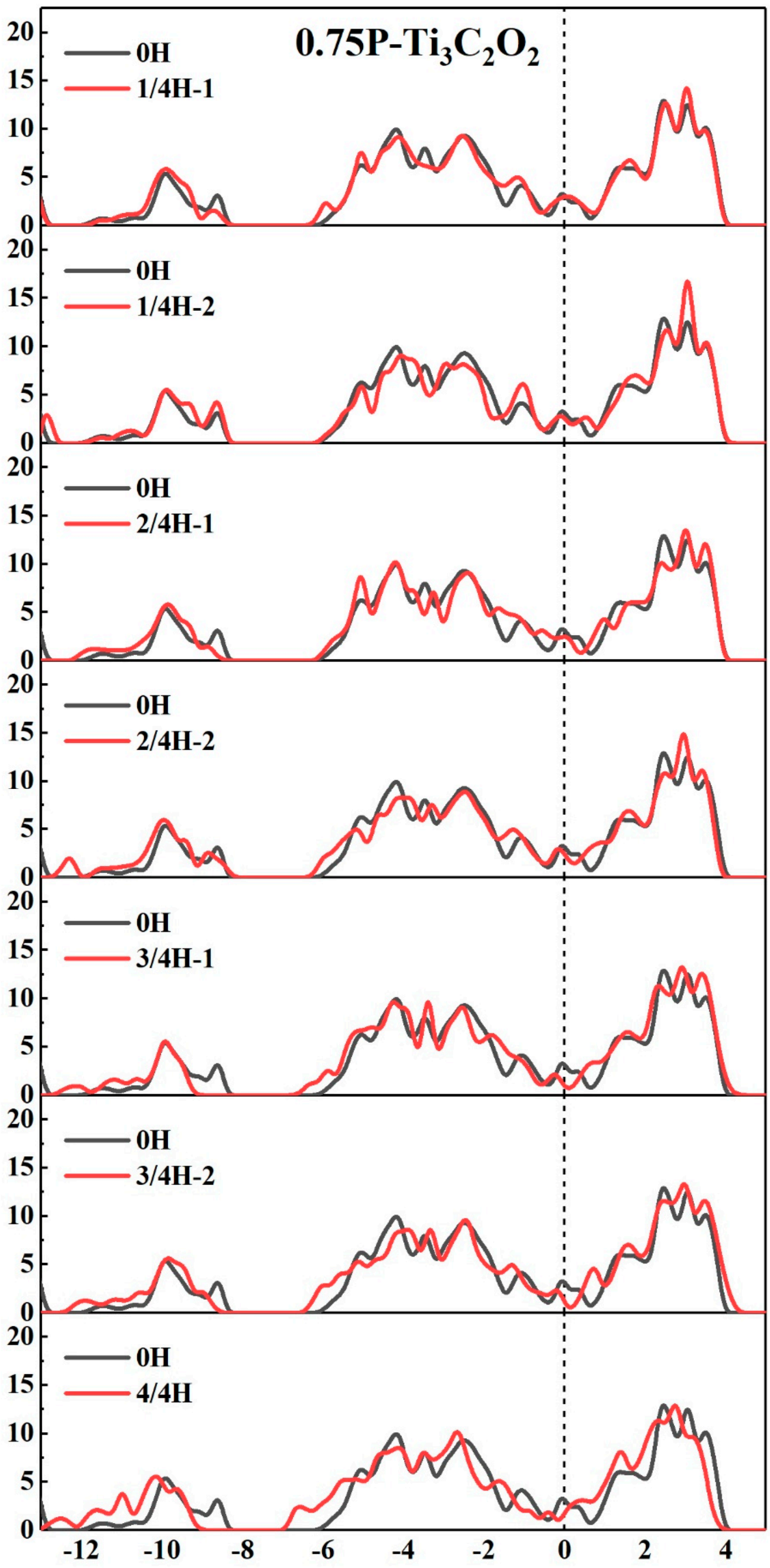

0.75P-$Ti_3C_2O_2$
0H
1/4H-1
1/4H-2
2/4H-1
2/4H-2
3/4H-1
3/4H-2
4/4H

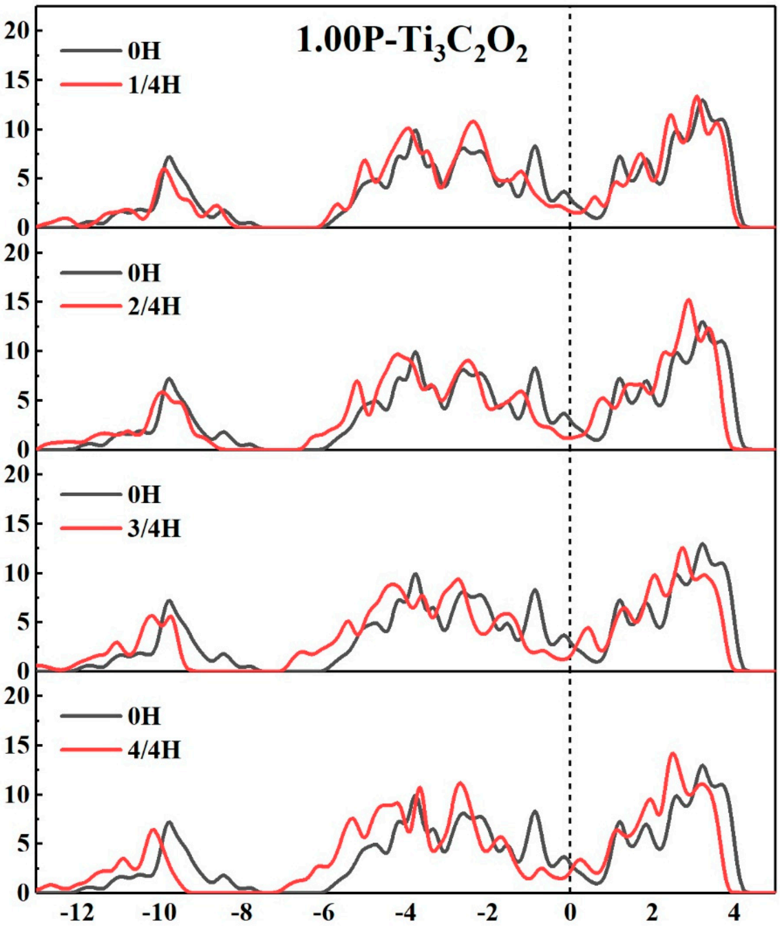

Figure S3. TDOS of different concentrations P element at different H element absorption concentrations. The black lines represent H atoms does not absorbed on the surface, the red lines represent H atoms absorbed on the surface.

Table S1. The hydrogen adsorption free energy(ΔG_H) of nonmetal doping $Ti_3C_2O_2$. 1-NM, 2-NM, 3-NM and 4-NM represent 1, 2, 3 and 4 H atoms absorbed on the doping nonmetallic atoms; 1-O, 2-O, 3-O represent 1, 2 and 3 H atoms absorbed on the surface O atoms. Bold values selected in the paper.

Doping Concentration	H coverage	configuration	ΔG(H)/eV Doping Element C	F	N	P	S
25%	1/4	1-NM	–1.387	–1.532	–1.035	–0.377	0.552
		1-O	–4.230	–3.705	–0.269	–0.308	–0.237
	1/2	1-NM,1-O	–0.792	–3.324	–0.530	–0.256	0.173
		2-O	–0.271	–3.324	–0.089	–0.205	–0.112
	3/4	1-NM,2-O	–0.512	–1.233	–0.270	–0.142	0.298
		3-O	–0.081	–3.054	0.077	–0.084	0.060
	1	1-NM,3-O	–0.284	–2.509	–0.028	0.034	0.384
50%	1/4	1-NM	–1.635	–3.448	–1.346	–0.908	0.520
		1-O	–0.571	–3.105	–0.526	–0.959	–0.304
	1/2	2-NM	–1.468	0.467	–1.170	–0.524	0.567
		2-O	–0.441	0.466	–0.314	–0.349	–0.635
		1-NM,1-O	–1.018	1.547	–1.327	–0.406	0.284
	3/4	2-NM,1-O	–1.065	-	–0.768	–0.337	0.530
		1-NM,2-O	–0.724	1.151	–0.458	–0.303	0.314
	1	2-NM,2-O	–0.821	1.539	–0.495	–0.190	0.586
75%	1/4	1-NM	–5.521	0.488	–1.854	0.149	0.556
		1-O	–4.652	0.488	–0.941	–0.086	–0.284
	1/2	2-NM	–5.193	2.878	–1.540	0.092	0.719

		**1-NM,1-O**	−4.719	1.507	−1.124	0.076	0.278
	**3/4**	**3-NM**	−5.934	1.000	-	0.012	0.904
		**2-NM,1-O**	−5.777	2.233	−1.072	0.034	0.612
	**1**	**3-NM,1-O**	−1.233	2.011	-	0.117	-
**100%**	**1/4**	**1-NM**	−6.704	−0.879	−2.132	−0.303	3.640
	**1/2**	**2-NM**	−4.637	−3.270	−1.924	−0.132	2.247
	**3/4**	**3-NM**	−3.363	−2.432	−3.222	0.008	1.927
	**1**	**4-NM**	−2.665	−0.894	−0.329	0.119	1.833

Table S2. Doped structure lattice constant, doping atomic radius and O-H bond length of 2D $Ti_3C_2O_2$ and nonmetal doping $Ti_3C_2O_2$.

NM	concentration	a	r	b(NM-Ti)
**O**	**-**	6.028	0.740	1.968
**B**	**0.25**	6.055	0.810	2.204
	**0.5**	6.060	0.810	2.209
	**0.75**	6.025	0.810	2.129
	**1**	6.000	0.810	2.285
**C**	**0.25**	6.063	0.770	1.982
	**0.5**	6.080	0.770	2.016
	**0.75**	6.072	0.770	2.013
	**1**	6.028	0.770	2.092
**F**	**0.25**	6.032	0.720	2.140
	**0.5**	6.036	0.720	2.125
	**0.75**	6.038	0.720	2.117
	**1**	6.040	0.720	2.149
**N**	**0.25**	0.740	6.048	1.921
	**0.5**	0.740	6.068	1.901
	**0.75**	0.740	6.076	1.923
	**1**	0.740	6.093	1.937
**P**	**0.25**	1.100	6.071	2.393
	**0.5**	1.100	6.083	2.482
	**0.75**	1.100	6.038	2.581
	**1**	1.100	6.005	2.608
**S**	**0.25**	1.040	6.058	2.391
	**0.5**	1.040	6.079	2.393
	**0.75**	1.040	6.093	2.399
	**1**	1.040	6.114	2.392
